# Benchmarking raw datasets and collaboratively-evolving processed data for markerless motion capture analysis

**DOI:** 10.1016/j.dib.2025.112044

**Published:** 2025-09-12

**Authors:** Antoine Muller, Alexandre Naaïm, Raphaël Dumas, Thomas Robert

**Affiliations:** Univ Lyon, Univ Gustave Eiffel, Univ Claude Bernard Lyon 1, LBMC UMR_T 9406, F-69622 Lyon, France

**Keywords:** Biomechanics, Kinematics, Marker-based, Video-based analysis, Joint angles

## Abstract

We present a dataset designed for benchmarking markerless motion capture methods (from videos to joint kinematics). The dataset includes both raw and processed data. Two participants performed five tasks - walking, sit-to-stand, manual material handling, handstand hold or Y-pose (depending on the participant), and a jointly performed dance sequence. Movements were captured simultaneously recorded using 10 optoelectronic cameras (Qualisys Miqus M3, 120 Hz) and 9 video cameras (Qualisys Miqus Video, 60 Hz, 1920×1088 pixels). The raw dataset provides 3D marker trajectories and video recordings. The processed dataset includes joint kinematics obtained from both marker-based motion capture and 7 different markerless methods, contributed by multiple research teams as part of a challenge organized during a national biomechanics seminar. Additionally, the open-access GitHub repository containing processed data enables researchers to contribute new markerless methods estimated and expand the dataset collaboratively. This resource aims to facilitate benchmarking and support the development of robust markerless motion analysis methods.

Specifications Table

**Table:** 

Subject	Engineering & Materials science
Specific subject area	Markerless motion capture analysis in biomechanics
Type of data	**Raw:** Video recordings, 3D marker trajectories. **Processed:** joints kinematics estimated using 7 different markerless motion capture methods; joint kinematics estimated using marker-based method.
Data collection	Two participants performed five tasks. Movements were simultaneously recorded using 10 optoelectronic cameras (Qualisys Miqus M3, 120 Hz) and 9 video cameras (Qualisys Miqus Video, 60 Hz, 1920×1088 pixels). Based on this raw data, 10 different joints angles were estimated using both marker-based motion capture and 7 different markerless motion capture methods. These markerless estimates were contributed by multiple research teams as part of a challenge organized during a national biomechanics seminar.
Data source location	Univ Lyon, Univ Gustave Eiffel, Univ Claude Bernard Lyon 1, LBMC UMR_T 9406, F-69,622 Lyon, France
Data accessibility	Repository name: Benchmarking dataset for markerless motion capture analysis Data identification number: https://doi.org/10.57745/LQI2MJ and https://doi.org/10.5281/zenodo.15730244 Direct URL to data: https://doi.org/10.57745/LQI2MJ and https://github.com/lbmc-lyon/Benchmarking_markerless Instructions for accessing these data: For **raw data**, to request access to the restricted data, please follow these steps on the website (https://doi.org/10.57745/LQI2MJ): 1. Download the Data Use Charter document. 2. Read it carefully, sign it, and send the signed version to antoine.muller@univ-lyon1.fr and thomas.robert@univ-eiffel.fr. 3. On the website, submit a request for access to the data. An example of anonymized data (gait trial of participant_02) is freely available for preview. For **processed data**, all is freely available on the Github repository.
Related research article	

## 1 Value of the Data

• The dataset enables benchmarking of markerless motion analysis methods through: 1) comparison with a marker-based motion analysis method, and 2) comparison among 7 different markerless methods.
• The dataset includes a variety of movements for evaluating classical motion analysis tasks, as well as more challenging tasks for markerless methods.
• The evolving benchmarking platform allows researchers to contribute to the dataset by integrating their own markerless motion analysis methods.

## 2 Background

Markerless motion analysis is gaining significant interest in biomechanics due to its potential applications, particularly the advantage of not requiring participant instrumentation. Various software solutions and methods have been developed to estimate biomechanical variables from video data [[1], [2], [3]]. Typically, these methods are evaluated by comparing their performance to marker-based assessments using datasets specifically designed by the authors for the purpose of the study [[4], [5], [6], [7], [8], [9]]. Several datasets dedicated to this type of evaluation are provided in the literature [[10], [11], [12]]. However, none of these datasets include processed markerless results, which makes it difficult to compare methods with each other. By creating a minimal yet sufficiently diverse dataset that includes raw video data, marker-based information, and estimates from various markerless methods, this new resource addresses the gap. It provides researchers with the necessary tools to conduct more thorough evaluations, facilitating a clearer understanding of each method's performance relative to others.

## 3 Data Description

**Fig. 1:**
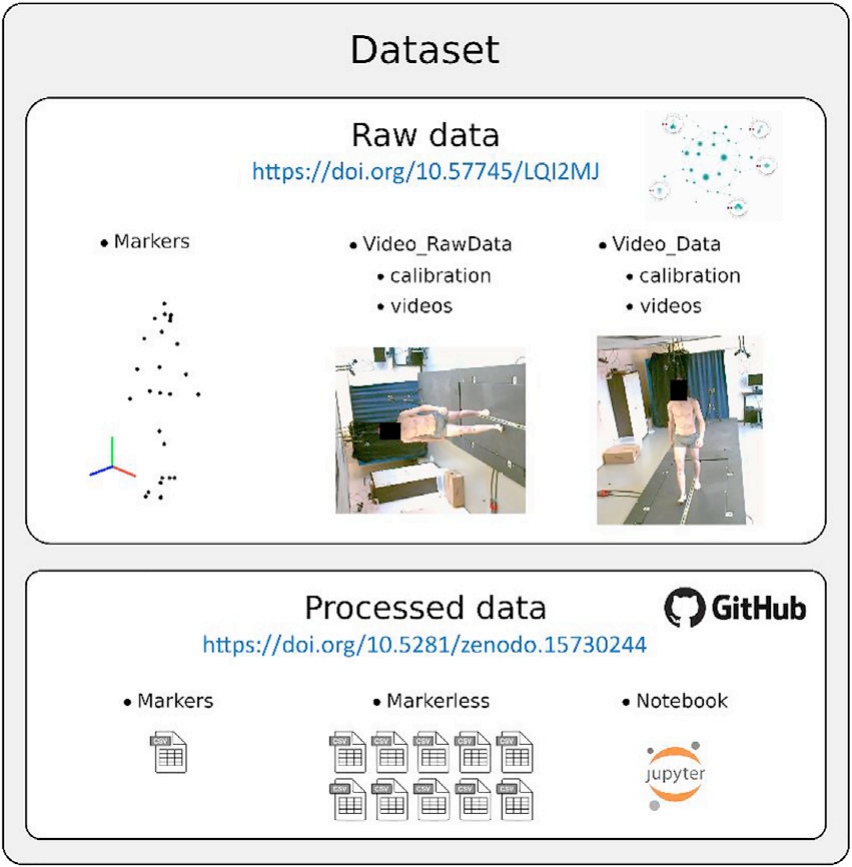
Illustration of the structure of the dataset.

The dataset is divided in two components (Fig. 1): i) **Raw data**: A data repository containing experimental data (3D marker trajectories and videos recordings); ii) **Processed data**: A GitHub repository, an evolving platform allowing researchers to contribute to the dataset by integrating their own markerless motion analysis methods. Initially, it includes joint kinematics estimates obtained using 7 different markerless methods (with a description of each computation model and algorithm) and joint kinematics estimates obtained using marker-based method.

### 3.1 Raw data

The repository contains 3 main folders:

• Markers: contains markers data. The structuration of this folder is detailed in Table 1.

**Table 1:** Structure of the Markers folder in the repository containing raw data.

Folder	File
/participant_01	/task1.c3d
	/task2.c3d
	…
/participant_02	/task1.c3d
	/task2.c3d
	…
• Video_RawData: contains raw video data and the cameras calibration file performed using Qualysis Track Manager. The structuration of this folder is detailed in Table 2.

**Table 2:** Structure of the Video_RawData folder in the repository containing raw data.

Folder	File
/calibration	/Calib.qca.txt
/both	/task1_Miqus_camNumber_camID.avi
	…
/participant_01	/task2_Miqus_camNumber_camID.avi
	…
/participant_02	/task2_Miqus_camNumber_camID.avi
	…
• Video_Data: contains pre-processed video data. Compared to Video_RawData, the videos in this folder have been rotated to ensure that the participants are correctly oriented. Consequently, the calibration file was modified to account for this rotation and converted into a ‘toml’ file using the OpenCV library notation. The structuration of this folder is detailed in Table 3.

**Table 3:** Structure of the Video_Data folder in the repository containing raw data.

Parent folder	Folder	File
/calibration		/Calib.toml
	/Checkerboard-intrinsic/videos/camID	/camID.avi
		…
	/Checkerboard-extrinsic-static/videos/camID	/camID.avi
		…
	/Checkerboard-extrinsic-dynamic/videos/camID	/camID.avi
		…
/Participant • /both • /participant_01 • /participant_02	/task1/videos/camID/	/camID.avi
		…
	/task2/videos/camID/	/camID.avi
		…
	…

### 3.2 Processed data

The processed data is available in a Github repository and include: i) a sample Jupyter Notebook code (compare_results.ipynb) to compare the different results; ii) markerless estimates of 10 joint angles using several methods; iii) marker-based estimates of the same 10 joints motions.

Markerless and marker-based data is organized within a main folder named ‘Data’, subdivided into two subfolders: ‘Markerless’ and ‘Markers’ (Table 4). As multiple methods were used for the markerless estimations, each method has its own folder within the ‘Markerless’ directory. For both markerless and marker-based methods, if the joint motion is estimated, a separate CSV file is provided for each participant and each task. These files are named following the pattern ‘task_participant.csv’. Regardless of the method, all CSV files share the same structure, which is detailed in the template available at ‘Data/Markerless/Template’. Time data are expressed in seconds, and joint motions are expressed in radians.

**Table 4:** Structure of the Github repository containing processed data.

Parent folder	Folder	File
		/compare_results.ipynb
Data/Markerless	Method1	Summary.xlsx
		task1_participant_01.csv
		…
	Method2	Summary.xlsx
		task1_participant_01.csv
		…
	…	
	Template	Summary.xlsx
		task_participant.csv
Data/Markers		task1_participant_01.csv
		…

**Fig. 2:**
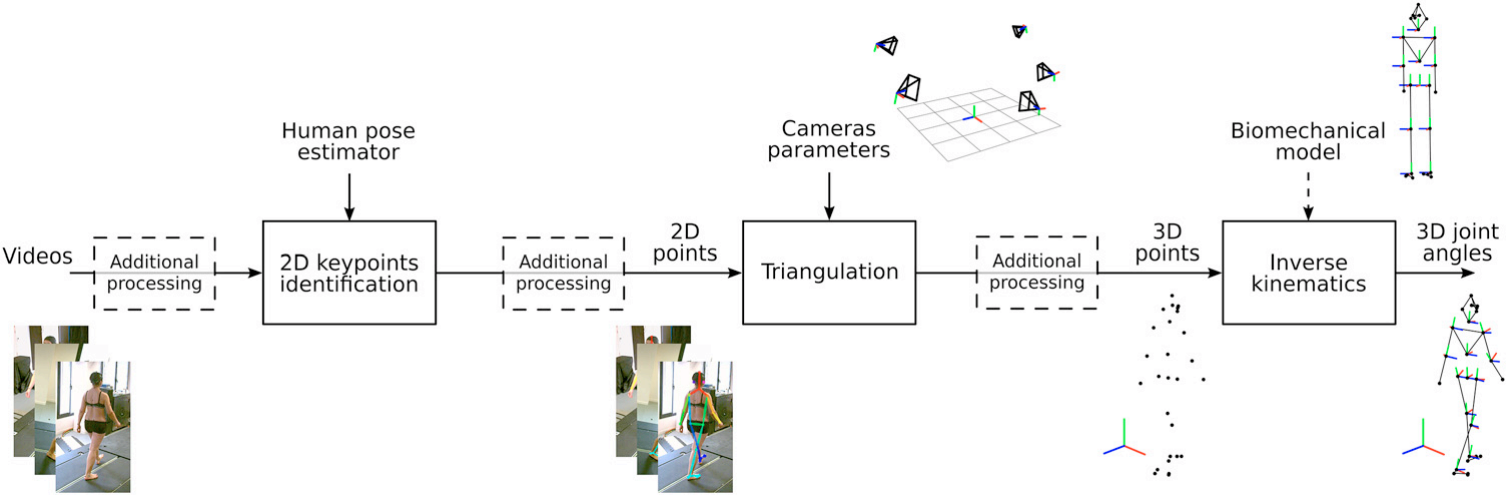
Workflow of the markerless motion analysis.

Additionally, each markerless method is accompanied by a ‘Summary’ file that describes both the method used to obtain the results and a summary table indicating which trials were processed and which were not. The description of the method is structured in a table, where each row corresponds to a step of the workflow illustrated in Fig. 2. The method used is described for each of these steps. A template of this file is also available at ‘Data/Markerless/Template’. The 10 estimated joints motions are (the names in brackets are the ones used in CSV files):

• right hip flexion/extension (‘RHip_FE’)
• right hip adduction/abduction (‘RHip_AA’)
• right knee flexion/extension (‘RKnee_FE’)
• right ankle flexion/extension (‘RAnkle_FE’)
• L5/S1 joint flexion/extension (‘L5S1_FE’)
• right shoulder adduction/abduction (‘RShoulder_AA)
• right shoulder internal/external rotation (‘RShoulder_RIE’)
• right elbow flexion/extension (‘RElbow_FE’)
• right elbow pronation/supination (‘RElbow_PS’)
• right wrist flexion/extension (‘RWrist_FE’)

## 4 Experimental Design, Materials and Methods

### 4.1 Experimental data

Two participants (P1: female, 29 years old, 157 cm, 60 kg; P2: male, 24 years old, 174 cm, 58 kg) were recruited for this experimental session. Prior to the experiment they signed an informed consent form. The study was approved by our institutional review board.

**Fig. 3:**
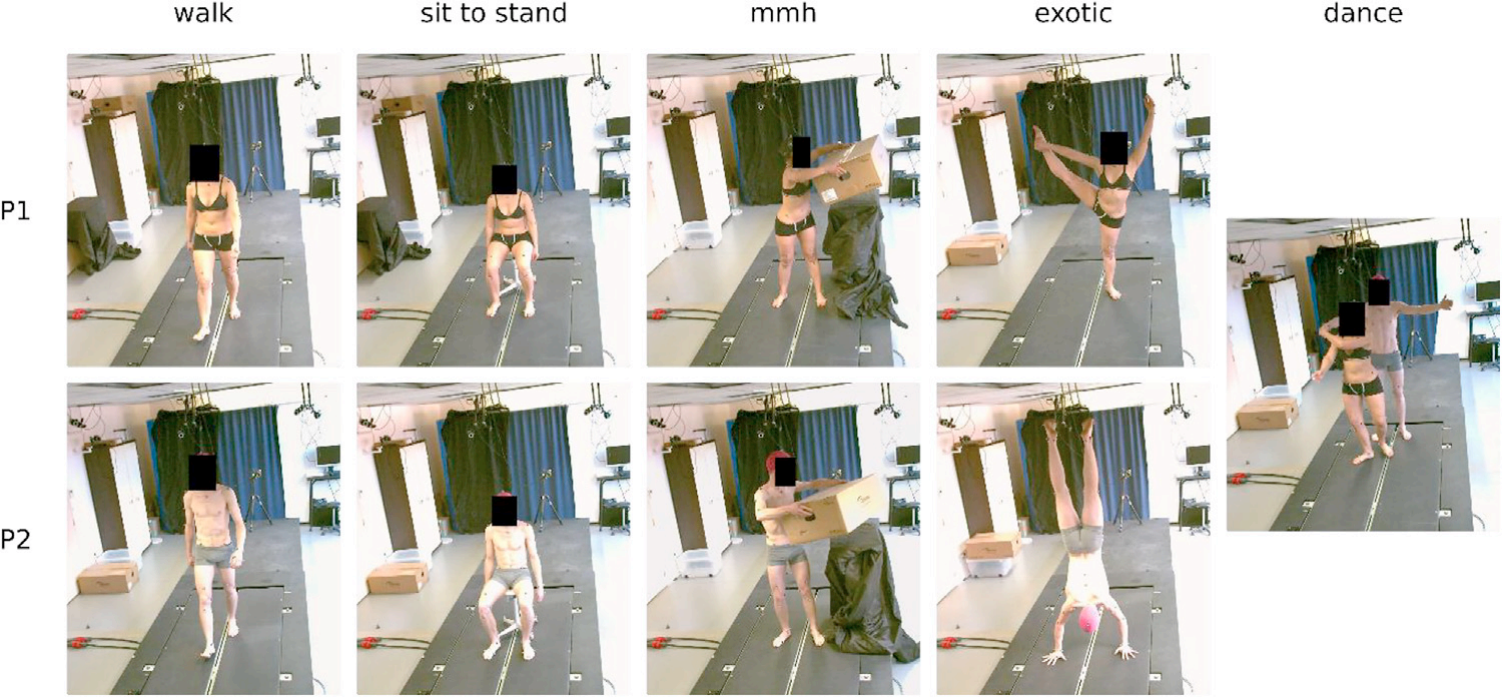
illustration of the five tasks performed by the two participants.

Both participants performed five tasks, illustrated in Fig. 3:

• ‘walk’: walk at self-selected comfort speed on a treadmill for about 20 cycles;
• ‘sit to stand’: sit to stand to sit on a stool;
• ‘mmh’: handling an empty cardboard box: the box is placed on the floor in front of the participant, who must pick it up and place it on a support to his left (at a height of about 1.2 m), then pick it up and place it back in its original position;
• ‘exotic’: the male participant performed handstand hold, the female participant performed a Y-pose;
• ‘dance’: about 15 s of dance performed together.

The tasks were simultaneously recorded by 10 optoelectronic (Qualisys Miqus M3, 120 Hz) and 9 video cameras (Qualisys Miqus Video, 60 Hz, resolution 1920×1088 pixels). Both systems were synchronized and calibrated using the Qualisys process (Qualisys Track Manager (QTM), v2021.1.2). Several videos with a checkerboard (7 × 4 verticies, 60 mm squares) were also captured to allow for cameras calibration.

The results of the experimental data constitute the **raw data**.

### 4.2 Markerless processing

Ten different markerless methods were applied to the same video data as part of a challenge organized during a national seminar dedicated to markerless motion capture. Using only markerless data, each participating team implemented their chosen methods and was tasked with estimating the 10 joints angles described above, for each task and each participant.

These estimations constitute a part of the **processed data**. Each markerless method was detailed on each corresponded ‘Summary’ file.

### 4.3 Marker-based processing

Markers trajectories were labeled and gap-filled using a traditional approach in QTM. A biomechanical model consisting of 16 segments -including the pelvis, abdomen, thorax, head, upper arms, forearms, hands, thighs, shanks, and feet- was developed based on a static trial, following the framework of [13]. Joint angles were then calculated using multibody kinematic optimization [14], allowing six degrees of freedom for each joint. This processing was performed using Pyomeca [15].

These estimations based on the maker-based motion capture constitute the other part of the **processed data**.

## Limitations

One limitation is that the dataset includes only two participants, each performing a single repetition of five tasks. Moreover, the participants are relatively homogeneous, being young and healthy, which may limit the generalizability of the benchmarking. However, although the dataset is minimal, it includes diverse body configurations (e.g., upside down) that can be considered challenging for most markerless methods. The limited number of participants also makes the dataset unsuitable for training new human pose estimation algorithms, although it can still be used to evaluate the performance of an already trained model.

## Ethics Statement

The authors confirm that prior to the experiment, participants signed an informed consent form. The study was approved by our institutional review board.

## CRediT Author Statement

**Antoine Muller:** Conceptualization, Software, Writing - Original Draft, Writing - Review & Editing, Project administration. **Alexandre Naaïm:** Conceptualization, Software, Investigation, Writing - Review & Editing. **Raphaël Dumas:** Conceptualization, Writing - Review & Editing. **Thomas Robert:** Conceptualization, Investigation, Project administration, Writing - Review & Editing.

## Data Availability

recherche.data.gouvBenchmarking dataset for markerless motion capture analysis (Original data). ZenodoBenchmarking platform for markerless motion capture analysis (Original data).
